# *Pluchea lanceolata* protects against Benzo(a) pyrene induced renal toxicity and loss of DNA integrity

**DOI:** 10.2478/intox-2013-0009

**Published:** 2013-03

**Authors:** Tamanna Jahangir, Mohammed M. Safhi, Sarwat Sultana, Sayeed Ahmad

**Affiliations:** 1 Department of Pharmacology, College of Pharmacy, Jazan University, Saudi Arabia; 2 Department of Medical Elementology and Toxicology, Hamdard University,India; 3 Department of Pharmacognosy, Hamdard University, India

**Keywords:** alkaline unwinding, B(a)P, DNA integrity, micronuclei, renal, quercetin

## Abstract

Evidence from epidemiological, experimental and clinical trial data indicates that a plant based diet can reduce the risk of chronic diseases and reduces toxic effects. In the present study, we report the antioxidant and anticlastogenic activity of *Pluchea lanceolata* (PL), an important medicinal plant, in both in vitro and *in vivo* model. Benzo(a)pyrene (B(a)P) administration leads to depletion of renal glutathione and its metabolizing enzymes. Pretreatment with PL (100 and 200 mg /kg b.wt) restored renal glutathione content and its dependent enzymes significantly (*p<*0.001) with simultaneous increase in catalase(CAT), quinone reductase(QR) in mouse kidney. Prophylactic administration of PL prior to B (a) P administration significantly decreased the malondialdehyde(MDA), H_2_O_2_ and xanthineoxidase (XO) levels at a significance of *p<*0.001, at both the doses. PL extract pretreated groups showed marked inhibition in B(a)P induced micronuclei formation in mouse bone marrow cells with simultaneous restoration of DNA integrity, viz. alkaline unwinding assay and DNA damage shown by gel-electrophoresis. HPTLC confirms the presence of quercetin in plant extract which could be responsible for PL protecting efficacy. In conclusion, the present findings strongly support the antioxidant efficacy of PL, possibly by modulation of antioxidant armory.

## Introduction

Benzo(a)pyrene (B(a)P), a polycyclic aromatic hydrocarbon, is a byproduct of combustion that causes renal injury and elicits a nephropathic response. Renal toxicity in rodents is often induced by B(a)P treatment (Alejandro *et al.*, 2000). Subchronic oral BaP dosing increased cast formation in Sprague Dawley rats (Knuckles *et al.*, 2001). Nanez *et al.* (2005) reported renal changes in female Sprague Dawley rats following once weekly injections of 10 mg/kg B(a)P for 16 weeks. B(a)P mediated alterations in the kidney affected predominantly to the glomerulus. An increase in micro albuminuria was seen in the B(a)P treated rats compared to controls (Nanez *et al.*, 2005). B(a)P is a pro-carcinogen, and its mechanism of carcinogenesis depends on its enzymatic metabolism to the ultimate mutagen, benzo(a)pyrene diol epoxide, a molecule that intercalates in DNA, covalently bonding to the nucleophilic guanine nucleic bases at the N2 position. X-ray crystallographic and nuclear magnetic resonance structure studies show that this binding distorts the DNA (Volk, 2003), inducing mutations by perturbing the double-helical DNA structure. This disrupts the normal process of copying DNA, induces mutations, and leads to the occurrence of cancer after exposure. This mechanism of action is similar to that of aflatoxin, which binds to the N7-position of guanine (Essigmannn, 1977).

There are indications that benzo[a]pyrene diol epoxide specifically targets the protective p53 gene (Shinmura, 2008). This gene is a transcription factor that regulates the cell cycle and hence functions as a tumor suppressor by inducing G (guanine) to T (thymidine) transversions within p53. There is a probability that benzo[a]pyrene diol epoxide inactivates the tumor suppression ability in certain cells, leading to cancer.

Identification and increased exposure to putative anti-mutagens may lead to a decreased rate of mutation and subsequently a decreased cancer incidence in humans (Stienmetz, 1991). The anticarcinogenic effect or function of natural compounds might be attributed to a combination of their cytoprotective effect on normal cells and their cytotoxic effect on preneoplastic and/or neoplastic cells. There is considerable scientific evidence to suggest that plant-based dietary factors can inhibit the process of many diseases effectively (Stienmetz, 1991). PL is an important medicinal plant, which is widely used in the traditional system of medicine for the cure of various diseases (Prasad, 1966). The decoction of PL has been used traditionally in curing arthritis (Chaturvedi, 1965). Quercetin and isorhamnetin were identified in air-dried leaves of PL (Chawla, 1991; Dixit, 1991).

Flavonoidrich diets are reported to have beneficial effects in cardiovascular diseases associated with overproduction of reactive oxygen species. They are reported to be scavengers of free radicals and potent inhibitors of lipid peroxidation (Zhou, 1991; Jimoh, 2007). These protective effects of flavonoids are chiefly ascribed to their antioxidant and vasodilator actions (Zenebe, 2001). Quercetin and isorhamnetin have been reported as active constituents of PL in previous studies (Chawla, 1991; Dixit, 1991). Quercetin (3,5,7,3’,4’-pentahydroxyflavone) is a flavone with putative ability to prevent cancer and cardiovascular diseases (Jones, 2004). Its metabolism was evaluated in rodents and humans. As acatechol, quercetin can potentially be converted to a quinone and subsequently conjugated with glutathione (GSH) (Jones, 2004). Results suggest that quercetin exerts its cancer-preventive effects by differential responses on mitogenic signaling and cell cycle regulators (Bhatia, 2001).

Isorhamnetin another active principle of PL, is the most potent inhibitor of CYP1B1d. This may be related to the more lipophilic substitution (O-methyl group) at the C-3’ position in the isorhamnetin molecule. Isorhamnetin, aflavonol aglycone, was investigated for its cytotoxicity and its influence on human hepatocellular carcinoma cells (BEL-7402) (Bao-song, 2006). The present study was designed to show the efficacy of PL against the toxic effects of B(a)P in an *in vivo* experimental model. The protective effects of PL may be attributed to the presence of its active ingredient, quercetin.

Our results support the assumption that enzymatic alterations may interpret the *in vivo* pharmacological effects, particularly in animal models and also in humans.

## Materials and methods

### Chemicals

Reduced glutathione (GSH), oxidized glutathione (GSSG), glutathione reductase, bovine serum albumin (BSA), 1,2,dithio-bis-nitrobenzoic acid (DTNB), 1,chloro-2,dinitrobenzene (CDNB), reduced nicotinamide adenine dinucleotide phosphate (NADPH), flavine adenine dinucleotide (FAD), glucose-6-phosphate, Tween-20,2,6,dichlorophenolindophenol and thiobarbituric acid (TBA) were obtained from SigmaChemical (St. Louis, MO, USA). All other chemicals and reagents were of the highest purity grade commercially available.

### Plant material

Total extract of PL in semi-solid form was procured from Saiba Industries Pvt. Ltd. It is claimed to possess all active principles of the plant.

### Animals

Swiss albino mice (20–25 g) were obtained from the Central Animal House Facility of Hamdard University, New Delhi and were housed in a ventilated room at 25±2°C under a 12-h light /dark cycle. The animals were acclimatized for one week before the *in vivo* study and had free access to standard laboratory feed (Hindustan Lever Ltd., Bombay, India) and water.

### Experimental design

For the study of biochemical parameters and micronuclei induction, eight-week-old adult male Swiss albino mice (20–25 g) were divided into five groups, each group consisted of five animals. B[a]P and PL were administered orally. B[a]P was administered in corn oil. In group I (vehicle control) the animals were given corn oil orally. The animals of group II served as treated control and were administered single oral dose of B(a)P (125 mg/g b.wt). The animals of group III were pretreated with 100 mg/kg b.wt of PL, while group IV and V were given 200 mg/kg b.wt of PL for seven consecutive days. The above-mentioned doses of PL were selected based on preliminary studies carried out in our laboratory (data not shown). On day 8, the animals of group II, III and IV were administered a single oral dose of B(a)P.

### Post-mitochondrial supernatant and microsome preparation

Tissue processing and preparation of post-mitochondrial supernatant (PMS) were done as described by Athar and Iqbal (1998). Kidneys were removed quickly, cleaned free of extraneous material and immediately perfused with ice-cold saline (0.85% sodium chloride). The kidneys were homogenized in chilled phosphate buffer (0.1 M, pH 7.4) containing KCI (1.17%) using a Potter Elvehjen homogenizer. The homogenate was filtered through muslin cloth and was centrifuged at 800 × g for 5 min at 4°C by Eltek Refrigerated Centrifuge (model RC 4100 D) to separate the nuclear debris. The aliquot so obtained was centrifuged at 12 000 rpm for 20 min at 4 °C to obtain post-mitochondrial supernatant **(**PMS), which was used as a source of enzymes. A portion of the PMS was centrifuged for 60 min by ultracentrifuge (Beckman L7-55) at 34 000 rpm at 4 °C. The pellet was washed with phosphate buffer (0.1 M, pH 7.4) containing KCI (1.17%). All the biochemical determination were completed within 24 h of animal sacrifice.

### Catalase activity

Catalase activity was measured by the method of Claiborne (1975). The reaction mixture consisted of 2 ml phosphate buffer (0.1M, pH 7.4), 0.95 ml hydrogen peroxide (0.019 M) and 0.05 ml PMS in a final volume of 3 ml. Changes in absorbance were recorded at 240 nm. Catalase activity was calculated as nmol H_2_O_2_ consumed per min/mg protein.

### Assay for glutathione-S-transferase activity

Glutathione-S-transferase activity was assayed by the method of Habig (1974). The reaction mixture consisted of 1.475 ml phosphate buffer (0.1 M, pH 6.5), 0.2 ml reduced glutathione (1 mM), 0.025 ml CDNB (1 mM) and 0.3 ml PMS (10% weight/volume (w/v) in a total volume of 2.0 ml. The changes in the absorbance were recorded at 340 nm and enzyme activity was calculated as nmol CDNB conjugate formed per minute per mg protein using a molar extinction coefficient of 9.6 × 10^6^/M/cm.

### Assay for glutathione peroxidase activity

Glutathione peroxidase (GPx) activity was measured by the method of Mohandas *et al.* (1984).

The reaction mixture consisted of 1.44 ml phosphate buffer (0.1 M, pH 7.4), 0.1 ml EDTA (1 mM), 0.1 ml sodium azide (1 mM), 0.05 ml glutathione reductase (1 IU/ml), 0.05 ml reduced glutathione (1 mM), 0.1 ml NADPH (0.2 mM), 0.01 ml H_2_O_2_ (0.25 mM) and 0.1 ml 10% PMS in a total volume of 2 ml. The disappearance of NADPH at 340 nm was recorded at 25 °C.

Enzyme activity was calculated as nmol NADPH oxidized/min/mg protein using a molar extinction coefficient of 6.22 × 10^3^ /M/cm.

### Assay for quinone reductase activity

The activity of quinone reductase was determined by the method of Benson (1980). The 3 ml reaction mixture consisted of 2.13 ml Tris–HCl buffer (25 mM, pH 7.4), 0.7 ml BSA, 0.1 ml FAD, 0.02 ml NADPH (0.1 mM), and 50 ml (10%) PMS. The reduction of dichlorophenolindophenol (DCPIP) was recorded calorimetrically at 600 nm and enzyme activity was calculated as n moles of DCPIP reduced per minute per mg protein using the molar extinction coefficient of 2.1 × 10^4^ /M/cm.

### Estimation of reduced glutathione

Reduced glutathione was determined by the method of Jollow (1974). One-milliliter sample of PMS was precipitated with 1.0 ml of sulphosalicylic acid (4%). The samples were kept at 4 °C for one hour and then centrifuged at 1 200 × g for 20 min at 4 °C. The assay mixture contained 0.1 ml filtered aliquot, 2.7 ml phosphate buffer (0.1M, pH 7.4) and 0.2 ml DTNB (100 mM) in a total volume of 3.0 ml. The yellow color developed was read at 412 nm on a spectrophotometer.

### Glucose-6-phosphate dehydrogenase activity

The activity of glucose-6-phosphate dehydrogenase was assayed by the method of (Zaheer *et al.*, 1965). The reaction mixture consisted of 0.3 ml tris-HCl buffer (0.05 M, pH 7.6), 0.1 ml NADP (0.1 mM), 0.1 ml glucose-6-phosphate (0.8 mM), 0.1 ml MgCl_2_ (8 mM), 0.3 ml PMS and 2.4 ml distilled water in a total volume of 3 ml. The changes in absorbance were recorded at 340 nm and enzyme activity was calculated as nmol NADPH oxidized/min/mg protein using a molar extinction coefficient of 6.22 × 10^3^/M/cm.

### Estimation of protein

The protein concentration was determined in all samples by the method of Lowry (1964).

### DNA Isolation

DNA was extracted from approximately 500 mg of kidney tissue by homogenizing the tissue in 5 ml TNE buffer (50 mM Trisma, 100 mM EDTA, 0.5% SDS, pH 8.0) in a 2 ml ground glass homogenizer. Each sample was homogenized with 10 standardized strokes of the pestle to minimize any potential effect on DNA integrity introduced by the homogenization procedure. An equal volume of buffered phenol/chloroform/isoamyl alcohol (PCI) (25:24:1, v/v/v, pH 8.0) was then added to the sample. The sample was gently mixed and allowed to settle for 5 min. It was then centrifuged for 5 min at 13 000 rpm at 4 °C. The aqueous layer was transferred to a new micro centrifuge tube and PCI extraction was repeated. The aqueous layer was then digested by 5ml of RNAase (10 mg/ml) for 30 min at 37°C and the digest was extracted once by PCI and once by 500 ml of chloroform. DNA was precipitated from the resulting aqueous layer by adding 2 volumes of absolute ethanol and 1/10 volume of 3 M sodium acetate, pH 5.2. The sample was then centrifuged (13 000 rpm, 15 min), and the resulting pellet rinsed with 500 ml of 70% ethanol and air-dried. The amount of DNA was quantitated spectro-photometrically at 260 and 280 nm (Khan, 2005; Xu, 1999) 2 mg/ml of DNA sample was dissolved in 1 ml of TE buffer (10 mM Trisma, 1 mM EDTA) and subsequently used in the DNA alkaline unwinding assay.

### Alkaline unwinding assay

The procedure used alkaline unwinding was essentially the same as that outlined by Shugart (1988) with slight modifications. In the alkaline unwinding assay, the rate of transition of double stranded DNA (dsDNA) to single stranded DNA (ssDNA) under pre-defined alkaline denaturing condition was proportional to the number of breaks in the phosphodiester backbone and thus was used as a measure of DNA integrity. Bisbenzamide was used as a DNA-binding dye and from its fluorescence various types of DNA were quantitated. For the fluorescence determination of dsDNA, ssDNA and partially unwound DNA (au-DNA), three equal portions of diluted DNA sample were prepared. The amount of dsDNA was obtained from the fluorescence of a sample without any treatment, while ssDNA was determined from the sample that had been boiled for 30 min. Fluorescence of the DNA sample which had been subjected to alkaline treatment (pH 12.2) on ice for 30 min provided an estimate of the amount of auDNA. The fluorescence of initial or double-stranded DNA was determined by placing 100 mmol DNA sample, 100 ml NaCl (25 mM) and 2 ml SDS (0.5%) in a pre-chilled test tube, followed by addition of 3 ml 0.2 M potassium phosphate pH 9, and 3 ml bisbenzamide (1 mg/ml). The contents were mixed and allowed to react in darkness for 15 min to allow fluorescence to stabilize. The fluorescence of the sample was measured using a spectro-fluorimeter (Ex: 360 nm, Em: 450 nm). The fluorescence of single stranded DNA was determined as above but using the DNA sample that had already been boiled for 30 min to completely unwind the DNA. The amount of 0.50 ml NaOH (0.05 N) was rapidly mixed with 100 ml of the DNA sample in a pre-chilled test tube. The mixture was incubated on ice in darkness for 30 min (Khan, 2005) followed by rapid addition and mixing of 50 ml HCl (0.05 N). This was followed immediately by addition of 2 ml SDS (0.5%) and the mixture was forcefully passed through a 21 G needle six times.

Fluorescence of alkaline unwounded DNA sample was measured as described above.

Measurement of the alkaline unwounded sample was performed in triplicate and the average was reported. The ratio between double stranded DNA to total DNA (F-value) was determined as follows: F value = (auDNA – ssDNA)/ (dsDNA – ssDNA) where auDNA, ssDNA and dsDNA were the degrees of fluorescence from the partially unwound, single stranded and double stranded determinations, respectively. The F value was inversely proportional to the number of strand breaks present and thus could be used as an indicator of DNA integrity.

### Gel electrophoresis and DNA fragmentation

The sample was mixed with 10mL of loading solution (10 mM EDTA (pH 8.0), 1%(w/v) bromophenol blue and 40% (w/v) sucrose) preheated to 70 °C. The DNA samples were loaded onto a 1.8% (w/v) agarose gel and sealed with 0.8% (w/v) low melting point agarose. The DNA fragments were separated by electrophoresis at 25 V for 12 h at 4 °C in TBE buffer. The DNA was visualized using ethidium bromide and photographed.

### Micronucleus test

For this test, mice were sacrificed 24 h after treatment with single oral dose of B(a)P. Femur bones for bone marrow were collected for micronucleus assay and kidney tissue for enzymatic assay. The time of peak response of micronuclei induction was selected as the sacrifice time for mutagen. This was decided based on the preliminary assays. A preliminary assay was done to select an appropriate dose of toxicant, which did not suppress cell proliferation, in combination with the highest plant dose. The mouse bone marrow micronucleus test was carried out according to the method of Schimid (1975). The cells were smeared on glass slide, air-dried and then stained successively with May-Gruenwald and Giemsa stain. Per animal, 2000 polychromatic erythrocytes (PCEs) were scored to determine the frequency of micronucleated polychromatic erythrocytes (MNPCEs) and the ratio of PCE/NCE was counted in 200 normochromatic erythrocytes (NCEs). A total of 2 500–3 000 polychromatic erythrocytes (PCEs) were scored per animal by the same observer for determining the frequencies of micronucleated polychromatic erythrocytes (MnPCEs).

### Detection of quercetin in the sample by TLC

The presence of quercetin shown in Figure 3, was determined in the given sample using 25 mg/mL solution in methanol by thin layer chromatography. The sample and standard quercetin (1mg/mL) were spotted in the form of bands of width 5 mm with a Camag microliter syringe on pre-coated silica gel aluminium plate 60F-254 (5 × 10 cm with 0.2 mm thickness, E. Merck, Germany) using a Camag Linomat V (Switzerland) sample applicator. A constant application rate of 120 nl/s was employed and space between two bands was 15 mm. The mobile phase consisted of hexane: ethyl acetate: formic acid (40:20:2.5). Linear ascending development was carried out in twin through glass chamber saturated with the mobile phase. The optimized chamber saturation time for mobile phase was 20 min at room temperature. The length of chromatogram run was 80 mm.

### Statistical analysis

Differences between groups were analyzed using analysis of variance (ANOVA) followed by Dunnett's multiple comparisons test. All data points are presented as the treatment groups mean ± standard error of the mean (SEM).

## Results

Table 1 depicts the effect of PL pretreatment on B(a)P induced alterations in reduced glutathione(GSH) content and its redox cycle. Treatment with B(a)P alone resulted in the depletion of renal glutathione and reduction in the activities of glutathione-S-transferase and glutathione reductase significantly (*p<*0.001),while PL treated groups showed restoration of glutathione redox cycle enzymes and GSH levels.

**Table 1:** Effect of pretreatment of PL extract on B(a)P mediated depletion in reduced glutathione, glutathione S-transferase and glutathione reductase.

Treatment regimen	Reduced glutathione (nmol GSH / g tissue)	Glutathione-S-transferase (nmol CDNB conjugate formed /min / mg protein )	Glutathione reductase (nmol NADPH oxidized/min/mg protein)
Corn oil treated control	0.099±0.006	217.9±1.04	175.2±11.5
B(a)p alone	0.045±0.0004##	465.03±3.14##	119.2 ±22.9##
B(a)p + PL (D1)	0.058±0.001#	468.0±2.09#	151.5±2.17#
B(a)p + PL (D2)	0.095±0.001#	337.0±4.19#	158.0±21.0#
Only PL (D2)	0.046±0.006	210.0±4.19	174.1±11.9

Results represent mean ± S.E of five animal\group. Results significantly different from corn oil treated group (^##^ *p<*0.001). Results significantly different from B(a)P treated group (^#^ *p<*0.001); PL=*Pluchea lanceolata;* D1 and D2 = 100 and 200 mg/kg body weight.

The effect of prophylactic treatment with PL on B(a)P induced reduction in the activities of renal antioxidant enzymes, as shown in Table 2. B(a)P treatment alone caused reduction in the activities of renal antioxidant enzymes such as catalase, glutathione peroxidase, and glucose-6-phosphate dehydrogenase and quinine reductase as compared to control group. Treatment with PL at the lower dose of 100 mg/kg body weight and the higher dose of 200 mg/kg body weight caused recovery of the above enzymes significantly (*p<*0.001), as compared with the B(a)P treated group.

**Table 2:** Effect of pretreatment of PL extract on antioxidant enzymes on B(a)p administration in kidney of Swiss albino mice.

Treatment regimen	Catalase (nmol H_2_O_2_ consumed/min/mg protein)	Quinone reductase (nmoles dichloroindophenol reduced/min/mg protein)	Glucose-6-phosphate dehydrogenase (nM NADP reduced/min/mg protein)	Glutathione peroxidase (nmol NADPH oxidized/min/mg protein)
Corn oil treated control	77.6±26.7	68.8±40.4	98.5 ± 2.31	78.4±30.7
B(a)p alone	111.9±18.4##	31.2±4.16##	47.6 ± 2.6##	64.5 ± 6.5##
B(a)p + PL (D1)	43.4±4.65##	39.8±1.97#	51.9 ± 3.3#	67.7±9.85#
B(a)p + PL (D2)	34.9±3.15#	50.4±0.93#	65.2 ± 10.4#	69.0±25.4#
Only PL (D2)	23.8±1.86	56.8±24.3	97.1 ± 0.23	99.8±3.2

Results represent mean ± S.E of five animal\group. Results significantly different from corn oil treated group (^##^ *p<*0.001). Results significantly different from B(a)P treated group (^#^ *p<*0.001); PL=*Pluchea lanceolata;* D1 and D2 = 100 and 200 mg/kg body weight.

Table 3 shows that B(a)P treatment enhances the activity of xanthine oxidase and susceptibility of renal microsomal membrane for iron-ascorbate induced lipid peroxidation and H_2_O_2_, as compared to controls. PL treatment caused reduction in the activity of xanthine oxidase, H_2_O_2_ and renal microsomal lipid peroxidation significantly (*p<*0.001), as compared with the B(a)P treated group.

**Table 3:** Effect of pretreatment of PL extract on B(a)P induced stress on malondialdehyde (MDA), H_2_O_2_ and xanthine oxidase (XO) level in mouse kidney.

Treatment groups	XO (µg uric acid formed/min/mg protein)	MDA (nmol MDA/h/g tissue)	H_2_O_2_ (n moles H_2_O_2_/g tissue)
Corn oil treated control	0.49±0.004	7.2±0.02	217.9±1.04
B(a)p alone	0.67±0.017##	9.18±0.03##	485.03±3.14##
B(a)p +PL (D1)	0.60±0.007#	8.3±0.01#	468±2.09#
B(a)p +PL (D2)	0.46±0.06#	8.0±0.02#	337±4.19#
Only PL (D2)	0.51±0.005	7.65±0.06	210±4.19

Results represent mean ± S.E of five animal\group. Results significantly different from corn oil treated group (^##^ *p<*0.001). Results significantly different from B(a)P treated group (^#^ *p<*0.001); PL=*Pluchea lanceolata;* D1 and D2 = 100 and 200 mg/kg body weight.

**Figure 1:**
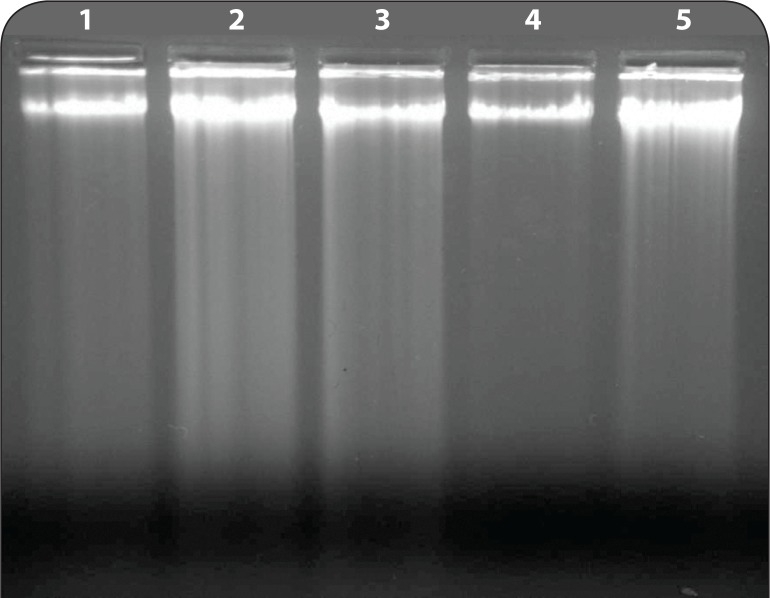
Agarose gel electrophoresis of DNA fragments in mouse kidney. DNA was isolated and assessed by agarose gel electrophoresis containing ethidiumbromide. 1 - only PL high dose, 2 - only toxicant, 3 - toxicant+PL low dose, 4 - control, 5 - toxicant+PL high dose

**Figure 2:**
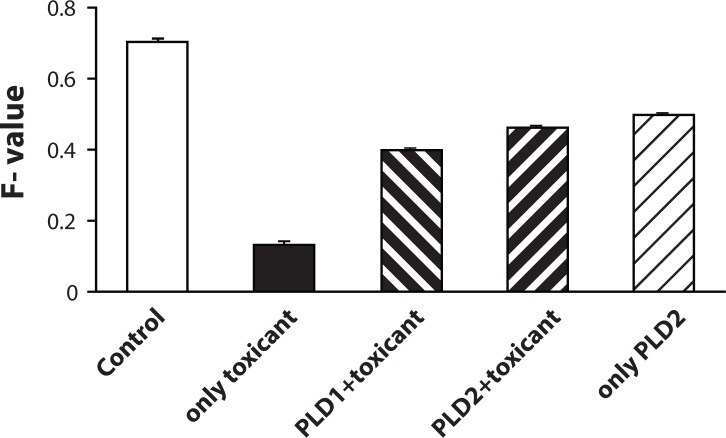
Effect of pretreatment of PL on B(a)P induced DNA damage in mouse (Alkaline unwinding assay). F value = (auDNA – ssDNA)/(dsDNA – ssDNA).

**Figure 3:**
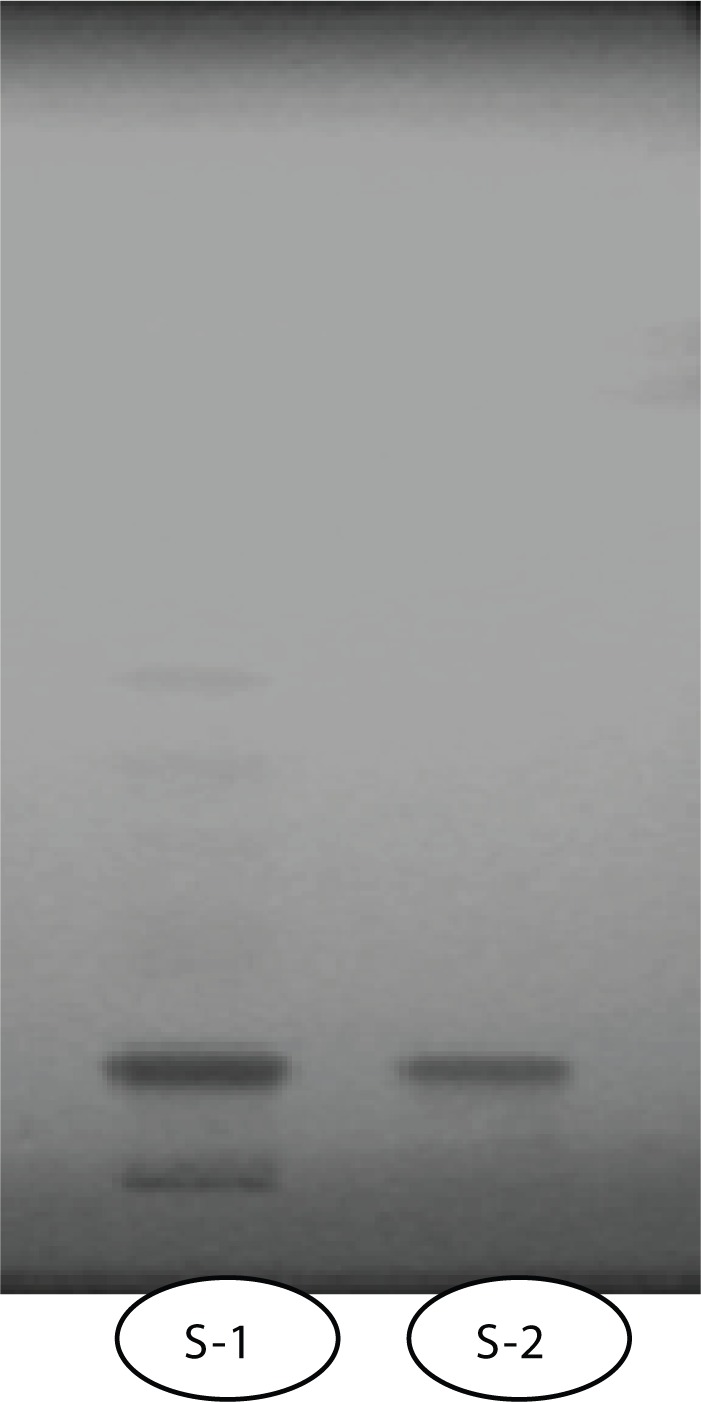
HPTLC plate pictograph showing presence of quercetin. S-1 =Sample; S-2=Standard quercetin

Table 4 illustrates the effect of pretreatment of the extract of PL on B(a)P induced micronuclei formation in mouse bone marrow cells where PL extract showed marked inhibition in micronuclei formation at both doses. Figure 1 shows agarose gel electrophoresis where results indicate that there was significant DNA fragmentation only in the B(a)P group as compared to control group and concurrent less fragmentation in PL pretreated groups at both doses. A simultaneous decrease in F-value was also noted in DNA alkaline unwinding assay as evident from Figure 2, which is a marker for alteration in DNA integrity. The F-value was inversely proportional to the number of strand breaks present, and thus has been used as an indicator of DNA integrity. During DNA fragmentation, DNA damage is estimated in terms of smearing and lack of intact bands. Control and only PL treated groups showed less smearing and an intact band. Figure 3 shows an HPTLC plate pictograph confirming the presence of quercetin in PL extract.

**Table 4:** Effect of pretreatment of PL extract on B(a)P induced micronuclei formation in mouse bone marrow cells.

Treatment regimen	Number of counted nucleated cells	Number of PE'S	Number of PE'S with micronuclei	% of PE'S ± SE	% of PE'S with micronuclei ± SE
Corn oil treated control	1870	339	21	18.1±4.1	6.1±0.96
B(a)p alone	1983	447	51	22.5±1.73*	11.4±0.52*
B(a)p + PL (D1)	1962	397	37	20.2±1.97 #	9.3±1.15#
B(a)p + PL (D2)	1906	378	31	19.8±2.78#	8.2±0.52#
Only PL (D2)	1923	364	20	18.9±3.8	5.5±0.74

No. of mice in each group was five and 1 500–3 000 nucleated cells were observed in each group.

* Results significantly different from corn oil treated group at *p<*0.01 level

# Results significantly different from B(a)p treated group at *p<*0.05 level.

## Discussion

Evidence from epidemiological, in vitro and in vivo studies indicates that a plant-based diet can reduce the risk of cancer and other chronic diseases (Rafter, 2002). The molecular mechanisms through which various nutrients might enhance or protect against carcinogenesis, the development of such biomarkers suitable for use in investigating the molecular effects of dietary factors in animal and human studies, and also in vitro studies, is of great importance. B(a) P is the most commonly studied mutagen with sources of exposure including occupation, diet and tobacco smoke (Phillips, 1999). The covalent binding of carcinogens to DNA is an important step in the cancer initiation process, with B(a)P requiring metabolic activation for DNA adduct formation to occur (Garner, 1998). The activation of carcinogens is primarily catalyzed by phase I enzymes, protection may be accomplished by inhibition of activating enzymes and/or by induction of phase II enzymes (Prochaska, 1988) which leads to detoxification and accelerated excretion of carcinogens. Several studies have shown that natural herbs and their compounds have potent antitumor promoting activities, possibly due to their antioxidant compounds (Jahangir, 2008; Wattenberg, 1997; Dhir, 1989; Hayatsu, 1988). PL extract ameliorated B(a)P-induced inhibition of the activities of the antioxidant enzymes glutathione peroxidase, glutathione reductase, catalase, glucose-6-phosphate dehydrogenase and of phase-II metabolizing enzymes such as glutathione-S-transferase and quinone reductase. PL has established antioxidant properties that might have counteracted the oxidant effects of B(a)P. The present study shows induction of renal glutathione-S-transferase and quinone reductase activity with PL prophylaxis treatment. The major mechanism for protecting against the toxic and neoplastic effects of carcinogens is the modification of cellular detoxification enzymes. Many environmental carcinogens require metabolism to reach their fully carcinogenic forms. They are often metabolized to proximate carcinogens by Phase I enzymes, like cytochrome P450, which catalyze oxidative reactions. The oxidized metabolites of carcinogenic compounds are then detoxified by Phase II metabolizing enzymes into the forms that are relatively less toxic and excretable (Bray, 1993; Zunino, 1989). Quinone reductase is a major enzyme of xenobiotic metabolism that carries out obligatory two-electron reductions and thereby protects cells against mutagenicity and carcinogenicity resulting from free radicals and toxic oxygen metabolites generated by the one-electron reductions catalyzed by cytochrome P450 and other enzymes. It has been shown that most of the chemopreventive agents result in the induction of glutathione-S-transferase and quinone reductase activity and in the degradation of electophilic metabolites. Induction of quinone reductase activity has been reported to have correlation with the prevention of cancer (De Flora & Ramel, 1988).

There was also dose-dependent decrease in the PL mediated susceptibility of renal microsomal membrane for iron-ascorbate induced lipid peroxidation through decreased production of free radicals as shown by depleted malondialdehyde levels. There was a decrease in the activities of xanthine oxidase, H_2_O_2_ and an increase in renal glutathione content.

There are large numbers of biomarkers available for assessing genotoxicity. Genomic instability is often measured as the characteristic of cancer. It has been shown earlier that B(a)P treatment leads to genotoxicity, chromosomal abbreviations,micronuclei induction DNA adduct formation, strand breaks, etc. in a rodent model of experiment (Khan, 2005). It is evident from this study that PL was not only able to reduce cellular damage but also suppressed DNA fragmentation and the formation of micronuclei polychlorinated erythrocytes MnPCEs in vivo, which are the hallmarks of B(a)P induced genotoxicity (Jahangir, 2008). Mouse bone marrow micronucleus assay is a widely used genotoxic assay to detect both clastogenic and aneugenic potencies of genotoxic agents or radiation (Ramalho *et al.*, 1991). Numerous epidemiological studies have suggested that chromosomal alterations including formation of micronuclei may serve as an effective biomarker to estimate cancer risk. In this study, it is evident from the results that PL reduced the number of micronuclei in the groups that were given PL + B(a)P in comparison with the B(a)P group. An increased number of micronuclei in PCE in comparison with the control group indicate that B(a)P produces chromosomal damage in erythrocytes of bone marrow and this damage is associated with the appearance and/or progression of tumors with adverse reproductive and developmental outcomes (Krishna & Hayashi, 2000).

From the presented findings, we can conclude that PL extract ameliorates B(a)P induced clastogenic effects in B(a)P treated mice. It is apparent from the present study that PL not only reduced cellular damage but also covered up the configuration of DNA integrity *in vivo* in PL pretreated groups in contrast to the only B(a)P group. There was a synchronous decline in the F-value in the DNA alkaline unwinding assay and differences observed with agarose gel electrophoresis, which are markers for DNA integrity. Our results show that there was significant DNA fragmentation in the B(a)P-treated group compared to the control group, whereas there was less fragmentation in the PL-pretreated groups. During DNA fragmentation, DNA damage is estimated by smearing and lack of an intact band on an agarose gel. An intact band was observed in the group pretreated with PL at dose II and in the control group only. The presented outcome gives direct confirmation that oxidative damage can be a major donor to DNA damage, which leads to reduction in F-value caused by B(a)P administration and simultaneously demonstrates the role of PL as a potent defense agent against B(a)P induced toxicity.

## Conclusion

Our data support the finding that PL has the efficacy of a potent antioxidant against B(a)P induced renal oxidative stress, loss of DNA integrity and micronuclei induction. The overall antioxidant and anticlastogenic efficacy of PL are probably due to the presence of flavanols like quercetin and isorhamnetin. They counteract with free radicals through their antioxidant mechanisms. Induction of antioxidant armory to suppress oxidative stress may be a possible mechanism of PL in modulating B(a)P toxicity. Thus inducers of antioxidant enzymes are potential candidate for preventive studies.
